# Supplementary material for: Global coordination of metabolic pathways in Escherichia coli by active and passive regulation
Source: Mol Syst Biol. 2021 Apr 14;17(4):e10064. doi: 10.15252/msb.202010064 (PMC8045939; doi:10.15252/msb.202010064)
Supplement: Supplementary file 1 — Appendix [file MSB-17-e10064-s003.docx]

**Appendix for**

**Global coordination of metabolic pathways in *Escherichia coli* by active and passive regulation**

Karl Kochanowski, Hiroyuki Okano, Vadim Patsalo, James Williamson, Uwe Sauer, Terence Hwa

Contents

[Appendix tables 2](#_Toc58776199)

[Appendix Text 3](#_Toc58776200)

[1. Strains used to genetically implement catabolic and anabolic limitation 3](#_Toc58776201)

[2. External titration of Crp activity 3](#_Toc58776202)

[3. Regulation analysis 4](#_Toc58776203)

[4. Relationship with phenomenological theory of bacterial growth control 6](#_Toc58776204)

[Appendix Figures 9](#_Toc58776205)

[References 28](#_Toc58776206)

# Appendix tables

**Appendix table S1. Strains used in this study.**

| **Strain name** | **Description** | **Source** |
| --- | --- | --- |
| NCM3722 | *Escherichia coli* K-12 wildtype strain. Parent of all strains used in this study | (Soupene *et al*, 2003) |
| NQ393 | *attB::* Φ(*Sp*:*lacI^Q^*:*tetR*) ∆*lacY* *∆gdhA* P*_Llac-O1_*:*gltBD*  IPTG inducible glutamate synthase (GOGAT) titration strain | (Hui *et al*, 2015) |
| NQ1243 | *zca-3633*::Φ(P_Ltet-O1_:*xylR*) Δ*ptsG468*::Φ(*kan*:P*u*)  3MBA inducible PtsG titration strain for upper range of PtsG expression. *ptsG* promoter is replaced with P*u* promoter. P_Ltet-O1_:*xylR* is inserted between *ycaC* and *ycaD*. | (Basan *et al*, 2015) |
| NQ1390 | *zca-3633*::Φ(*lacI^q^p*:*xylR*) Δ*ptsG468*::Φ(*kan*:P*u*)  3MBA inducible PtsG titration strain for lower range of PtsG expression. *ptsG* promoter is replaced with P*u* promoter. *lacI^q^p*:*xylR* is inserted between *ycaC* and *ycaD*. | this study |
| NQ1399 | Δ*cyaA::FRT,* Δ*cpdA::FRT* | this study |

# Appendix Text

## Strains used to genetically implement catabolic and anabolic limitation

All strains used in this study were derived from NCM3722 (Soupene *et al*, 2003) (list of used strains in supplementary table 1). To impose a gradual catabolic limitation while maintaining a constant cellular environment, we constructed strains in which the expression of a key component of the glucose uptake system, PtsG, is put under the control of the P*u* promoter from *Pseudomonas putida*, which in turn is activated by the regulator XylR upon induction by 3-methylbenzyl alcohol (3MBA) (Basan *et al*, 2015) (supplementary figure S1, left panel). Importantly, since different glycolytic carbon (i.e. glucose and lactose) titration strains show highly similar physiological behavior (Basan *et al*, 2015), they can be treated as physiologically equivalent.

Similarly, to impose a gradual anabolic limitation, we used a recently constructed strain in which the assimilation of the sole nitrogen source ammonium can be tuned externally. Specifically, we deleted the ammonium-assimilating enzyme glutamate dehydrogenase (GdhA), and put the expression of glutamate synthase (GOGAT) under the control of the synthetic P*lac* promoter from *E. coli.* GOGAT catalyzes the conversion of 2-oxoglutarate plus glutamine to two molecules of glutamate. By adding IPTG, we titrate the expression of GOGAT and thus the rate of glutamate formation (Hui *et al*, 2015) (supplementary figure S1, right panel).

## External titration of Crp activity

To construct a mutant strain that is incapable to endogenous cyclic AMP production and degradation, we deleted the genes encoding the enzymes adenylate cyclase (CyaA) and cyclic AMP phosphodiesterase (CpdA) in NCM3722 wild-type by P1 phage transduction using the respective deletion strains from the Keio collection (Baba *et al*, 2006) followed by excision of the selection marker as described previously (Basan *et al*, 2015). Thus, in this strain, termed NQ1399, the activity of Crp can be controlled externally both above and below wild-type level by supplementing the medium with varying concentrations of cyclic AMP (cAMP). We found that both diminished and gratuitous Crp activity impair the steady state growth rate, with a maximal growth rate at intermediate cyclic AMP concentrations that closely matches wild-type growth (supplementary figure S4). To test whether adenylate cyclase has a potential regulatory function beyond its role in cyclic AMP production, we compared the proteome of NQ1399 at the maximal growth rate (by adding 0.2 mM cAMP to the medium) with the NCM3722 wild-type (supplementary figure S7).

The vast majority of proteins showed little difference between NQ1399 and NCM3722. However, there were few notable exceptions, such as flagella and chemotaxis proteins, which were almost completely absent in NQ1399. A possible explanation for this observation could be that one of the deleted proteins has a non-canonical function (i.e. acting as a non-canonical transcription factor), or that they catalyze the production (or consumption) of other cyclic nucleotides which affect flagella/chemotaxis protein expression directly or indirectly. Since flagella and chemotaxis proteins account for a significant fraction of the catabolic proteome sector, we excluded these proteins (as well as all other proteins which showed more than four-fold differences between NCM3722 and NQ1399 with 0.2 mM cAMP) in further analyses.

Next, we quantified the proteome of NQ1399 at varying external cAMP concentrations (ranging between 0 and 3 mM cAMP) and compared the resulting proteome sector fractions with the re-calculated sector fractions from carbon and anabolic limitations (supplementary figure S8). For most sectors, the response to carbon limitation and high cAMP concentration (= gratuitous Crp activity compared to WT), as well as to anabolic limitation and low cAMP concentration (= reduced Crp activity compared to WT), showed good quantitative agreement. Notably, the overlap between carbon limitation and high cAMP concentration for anabolic proteins (sector A) was less pronounced, suggesting that in carbon limitation additional regulatory mechanisms, for example promoter specific transcriptional regulators, may at least partially compensate the indirect repression by Crp. For example, the two most abundant A-sector proteins, OmpA and ThrA, whose response to anabolic and catabolic limitation was poorly recapitulated by cAMP titration (supplementary figure S9), are both regulated posttranscriptionally by RNA or ribosome mediated attenuation, respectively.

Finally, we wanted to identify proteins which are not affected by the aforementioned global transcriptional program (that is, whose transcription is predominantly regulated by designated regulators, rather than indirect repression by Crp). In particular, we focused on proteins whose concentration strictly depends on the growth rate. Towards this end, we calculated the Pearson correlation between relative protein concentration and growth rate across limitations and cAMP titration experiments (supplementary figure S10). As expected, ribosomal proteins were among the proteins with the strongest positive correlation (supplementary figure S10B, top panel). Nevertheless, several non-ribosomal proteins, such as the sulfate assimilation proteins CysI and CysJ (supplementary figure S10B, middle panel), showed a similar response. In particular, MetE, which catalyzes the last step of methionine biosynthesis and is considered a highly “expensive” protein (Li *et al*, 2014), maintained a positive correlation with growth rate across all tested conditions, suggesting that its expression is finely tuned to match the growth dependent demand for methionine.

## Regulation analysis

To assess the impact of changes in enzyme and metabolite concentrations on steady state reaction rates, we use the recently developed ‘regulation analysis’ framework (Rossell *et al*, 2006; Chubukov *et al*, 2013; Gerosa *et al*, 2015). In this section, we provide a detailed derivation of the relevant equations following these previous works.

As a starting point, we use a recently published derivation of a reversible Michaelis-Menten type reaction (Noor *et al*, 2013) (**eq. S1**):

$$J=\left( E \cdot k_{cat} \right)\cdot\left( \frac{\frac{S}{K_{S}}}{1+\frac{S}{K_{S}}+\frac{P}{K_{P}}} \right)\cdot\left( 1-e^{\Delta G'/RT} \right)=P\cdot S \cdot D$$

where J denotes the reaction rate (or flux), E denotes the enzyme concentration, k_cat_ denotes the enzyme’s turnover number, S and P denote the enzyme’s substrate and product with their specific parameters K_S_ and K_P_, respectively, and ΔG’ denotes the reaction’s thermodynamic potential with Gas constant R and temperature T. The three terms P, S, and D capture the contribution of enzyme concentration, enzyme saturation, and thermodynamic driving force, respectively. This derivation can be further simplified by neglecting the (typically small) influence of the product on enzyme saturation and further approximating the saturation term by a power law term with a metabolite-specific parameter α, as described previously (Chubukov *et al*, 2013; Gerosa *et al*, 2015) (**eq. S2**):

$$J=\left( E \cdot k_{cat} \right)\cdot\left( \frac{S}{K_{S}} \right)^{\alpha}\cdot D$$

To simplify eq. S2 further, we can normalize the data to a reference condition (**eq. S3**):

$$\frac{J}{J_{ref}}=\left( \frac{E}{E_{ref}} \right)\cdot\left( \frac{S}{S_{ref}} \right)^{\alpha}\cdot\left( \frac{D}{D_{ref}} \right)$$

Upon transformation into log space (**eq. S4**):

$$\log\left( \frac{J}{J_{ref}} \right)=\log\left( \frac{E}{E_{ref}} \right)+\alpha\cdot\log\left( \frac{S}{S_{ref}} \right)+\log\left( \frac{D}{D_{ref}} \right)$$

Thus, changes in log normalized flux (relative to a reference condition) can be expressed by a linear combination of respective changes in log normalized protein and substrate concentration, as well as thermodynamic driving force. Dividing eq. S4 for log(J/J_ref_) then yields (**eq. S5**):

$$1=\frac{\log\left( \frac{E}{E_{ref}} \right)}{\log\left( \frac{J}{J_{ref}} \right)}+\frac{\alpha\cdot\log\left( \frac{S}{S_{ref}} \right)}{\log\left( \frac{J}{J_{ref}} \right)}+\frac{\log\left( \frac{D}{D_{ref}} \right)}{\log\left( \frac{J}{J_{ref}} \right)}= \rho_{P}+\rho_{S}+\rho_{D}$$

Where ρ_P_, ρ_S_, ρ_D_ and denote the protein, substrate, and driving force regulation coefficients. Importantly, eq. S5 illustrates that in absence of any additional regulation (such as allosteric regulation), these regulation coefficients have to sum up to 1. Thus, regulation analysis provides a means to – for every reaction - quantitatively assess the **consistency** between observed changes in flux and corresponding changes in enzyme and substrate concentrations, as well as the thermodynamic driving force.

To illustrate how to use this equation, let us consider the two scenarios examined in this work. In the first scenario, both enzyme saturation and thermodynamic driving force of a reaction remain constant across conditions (which is equivalent to an irreversible enzyme operating close to full saturation). In this case, eq. S5 simplifies to (**eq. S6**):

$$\frac{\log\left( \frac{E}{E_{ref}} \right)}{\log\left( \frac{J}{J_{ref}} \right)}= \rho_{P}=1$$

Such a reaction will exhibit consistent proportional changes in flux and protein concentration across conditions, and can be interpreted to be predominantly regulated transcriptionally (Daran-Lapujade *et al*, 2007).

If both enzyme and substrate concentration change across conditions, eq. S5 simplifies to (**eq. S7**):

$$\frac{\log\left( \frac{E}{E_{ref}} \right)}{\log\left( \frac{J}{J_{ref}} \right)}+\frac{\alpha\cdot\log\left( \frac{S}{S_{ref}} \right)}{\log\left( \frac{J}{J_{ref}} \right)}= \rho_{P}+\rho_{S}=1$$

In this case, changes in flux will be fully proportional with the combination of protein (captured by ρ_P_) and substrate (captured by ρ_S_) concentration changes. Note that eq. S7 can be easily expanded to account for multiple substrates (**eq. S8**):

$$\rho_{P}+\rho_{S}=\frac{\log\left( \frac{E}{E_{ref}} \right)}{\log\left( \frac{J}{J_{ref}} \right)}+\frac{\sum_{i}^{n} \alpha_{i}\cdot\log\left( \frac{S_{i}}{S_{i ref}} \right)}{\log\left( \frac{J}{J_{ref}} \right)}= 1$$

Note that in order to calculate ρ_S_, one needs to determine the metabolite-specific parameters α for each substrate of a given reaction. As described in previous works (Chubukov *et al*, 2013; Gerosa *et al*, 2015), these parameters can be estimated from flux, protein, and metabolite changes (see methods in main text).

## Relationship with phenomenological theory of bacterial growth control

Here, we discuss the relationship between the mechanistic insights revealed by this work and the predictability of the proteome responses according to the phenomenological theory of bacterial growth control introduced previously (Scott *et al*, 2010; You *et al*, 2013; Hui *et al*, 2015). It was shown empirically that the fractional abundances of catabolic and anabolic proteins, referred to as C- and A- proteins, and denoted as $\phi_{C}$ and $\phi_{A}$, respectively, exhibited approximately linear dependences on the growth rate, for cells subjected to catabolic and anabolic limitations. These empirical dependences, together with an overall constraint on proteome allocation, i.e. (**eq. S9**),

$$\phi_{C}+\phi_{A}+\phi_{R}=\phi_{\text{max}}$$

where $\phi_{R}$ is the fractional abundance of ribosomal proteins and affiliated translational machineries (referred to as R-proteins) and $\phi_{\text{max}}$ is a constant, allowed quantitatively accurate predictions on the growth-rate dependences of the abundances of the C-, A-, and R- proteins under catabolic and anabolic limitations and their combinations. The results of this study now allow us to examine the mechanistic origin of these empirical dependences.

The empirical dependences of the proteome fractions on the growth rate ($\lambda$) observed are as follows (**eq.’s S10a,** **S10b**):

$$\phi_{C}=\phi_{\text{max}}\cdot(1-\lambda/\lambda_{C})$$

$$\phi_{A}=\phi_{A,0}+\lambda/\nu_{A}$$

under catabolic limitation, and (**eq.’s S11a, S11b**):

$$\phi_{C}=\phi_{C,0}+\lambda/\nu_{C}$$

$$\phi_{A}=\phi_{\text{max}}\cdot(1-\lambda/\lambda_{C})$$

under anabolic limitation, with (**eq S12**):

$$\phi_{R}=\phi_{R,0}+\lambda/\nu_{R}$$

under both catabolic and anabolic limitations. The rationalization of the linear dependence of R-protein abundances on the growth rate has been extensively discussed previously (Maaløe, 1979; Scott *et al*, 2010). For the abundances of C- and A- proteins, it was previously theorized that eq.’s **S10b**, **S11A** were valid under both catabolic and anabolic modes of growth limitations. Catabolic limitation corresponds to changes in the value of $\nu_{C}$, for which eq. **S11A** cannot be used to describe the growth-rate dependence of C-protein abundances ($\phi_{C}$), and the latter is fixed by the combination of Eq.’s **S10**, **S12**, and the constraint **S9**, resulting in eq. **S10A**. Similarly, anabolic limitation corresponds to changes in the value of $\nu_{A}$, with eq. **S11b** resulting from the combination of eq.’s **S11A**, **S12**, and **S9**.

The mechanistic origin of Eq. **S11b** and **S10A** can be described as follows: Let the metabolic flux driven by the C- and A- proteins be $J_{C}$ and $J_{A}$ respectively. We can write these two fluxes as (**eq.’s S13, S14**)

$J_{C}=k_{C}\cdot(M_{C}-M_{C,0})$

$$J_{A}=k_{A}\cdot(M_{A}-M_{A,0})$$

where $M_{X}\equiv\phi_{X}\cdot M$ ($X\in\{C,A\}$) is the abundance of each type of proteins, $M$ being the total protein abundance. Taking each flux to be proportional to the growth rate, i.e. (**eq. S15**)

$$J_{X}=c_{X}\lambda M$$

where $c_{X}$ is related to the yield coefficient, the empirical relations **S11A** and **S10B** are recovered from eq.’s **S13** and **S14** with $M_{X,0}=\phi_{X,0}\cdot M$, and $\nu_{X}=k_{X}/c_{X}$. Since in minimal media eq. **S15** holds for the vast majority of metabolic fluxes (in particular outside central carbon metabolism, see main text), the origin of the observed relations **S10A**, **S10B**, **S11A**, **S11B** can be traced to the rationalization of eq.’s **S13** and **S14**.

If the constants $M_{C,0}$ and $M_{A,0}$ are zero, then eq.’s **S13** and **S14** simply express a linear relation between flux and the abundance of enzymes driving the flux, in the regime where the flux is completely limited by the enzyme abundances, or if the reaction substrates are completely saturating. The constants $M_{C,0}$ and $M_{A,0}$ are the simplest way to account for the sub-saturation effect by the metabolite pools, since they reduce their respective flux from the maximal saturated level. The results of Figures 3b and 3c (see main text) show that under catabolic limitation, the combination of enzyme abundance and enzyme saturation effects indeed give a reasonable account of the fluxes through A-proteins, thereby justifying eq. **S14** (hence eq. **S10B**). Under anabolic limitation, we know the abundances of the A-proteins are set passively, and thus do not expect eq. **S14** (hence eq. **S10B**) to hold. Instead, A-protein abundance is expected to be set through the C-proteins, via eq. **S13**.

For anabolic limitation with glucose as the sole carbon source, the only necessary catabolic protein used by *E. coli* is the PTS transporter PtsG, which indeed shows positive correlation between expression and growth under anabolic limitation (supplementary figure S17), presumably regulated by Crp. However, Crp also regulates the expression of many other catabolic proteins even if their substrates are not present (including many transporters, see supplementary figure S18, presumably reflecting the co-occurrences of these substrates in *E. coli*’s natural habitat), and further includes the flagella system (supplementary figure S19). Thus, we view eq. **S13** to reflect *E. coli*’s pre-programmed response to growth limitation by nutrients other than carbon.

# Appendix Figures


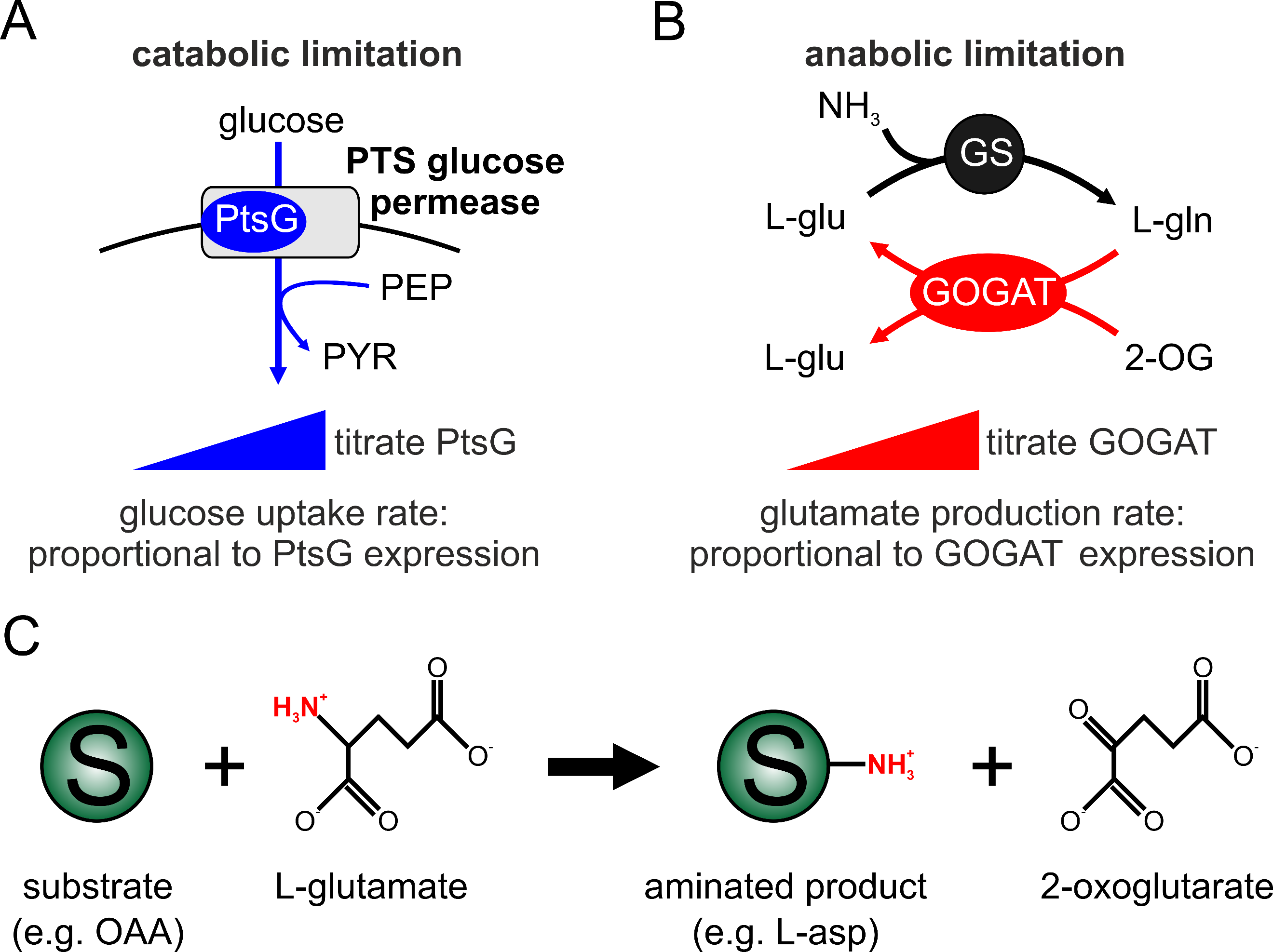


**Appendix figure S1. Schematic of genetically implemented limitations. A)** to mimic a limitation of carbon supply (equivalent to a carbon-limited chemostat), the glucose uptake system component PtsG was put under the control of the 3-MBA inducible promoter Pu from *Pseudomonas putida* (Basan *et al*, 2015). Thus, by titrating the expression of PtsG, the glucose uptake rate can be gradually adjusted, resulting in a catabolic limitation. **B)** To mimic a limitation of nitrogen assimilation (equivalent to a nitrogen-limited chemostat), the enzyme glutamate synthase (GOGAT, encoded by the *gltBD* operon) was put under the control of the IPTG inducible synthetic promoter P*lac* from *Escherichia coli* (Hui *et al*, 2015). The second nitrogen assimilating enzyme glutamate dehydrogenase (GDH, encoded by *ghdA*) was deleted. Thus, by titrating the expression of GOGAT, the rate of glutamate production can be gradually adjusted, resulting in a limitation of anabolic capacity to perform glutamate-dependent transamination reactions. **C)** Schematic of glutamate-dependent transamination reactions (using the biosynthesis of L-asp from PYR as an example), which are pivotal reaction steps in many amino acid biosynthesis pathways. Thus, lack of L-glu generally impairs the cell’s ability to produce biomass building blocks. PEP: phosphoenolpyruvate, PYR: pyruvate, L-Gln: L-glutamine, L-Glu: L-glutamate, 2-OG: 2-oxoglutarate. OAA: oxaloacetate, L-Asp: L-aspartate. See supplementary table 1 for the exact genotype of each used strain.


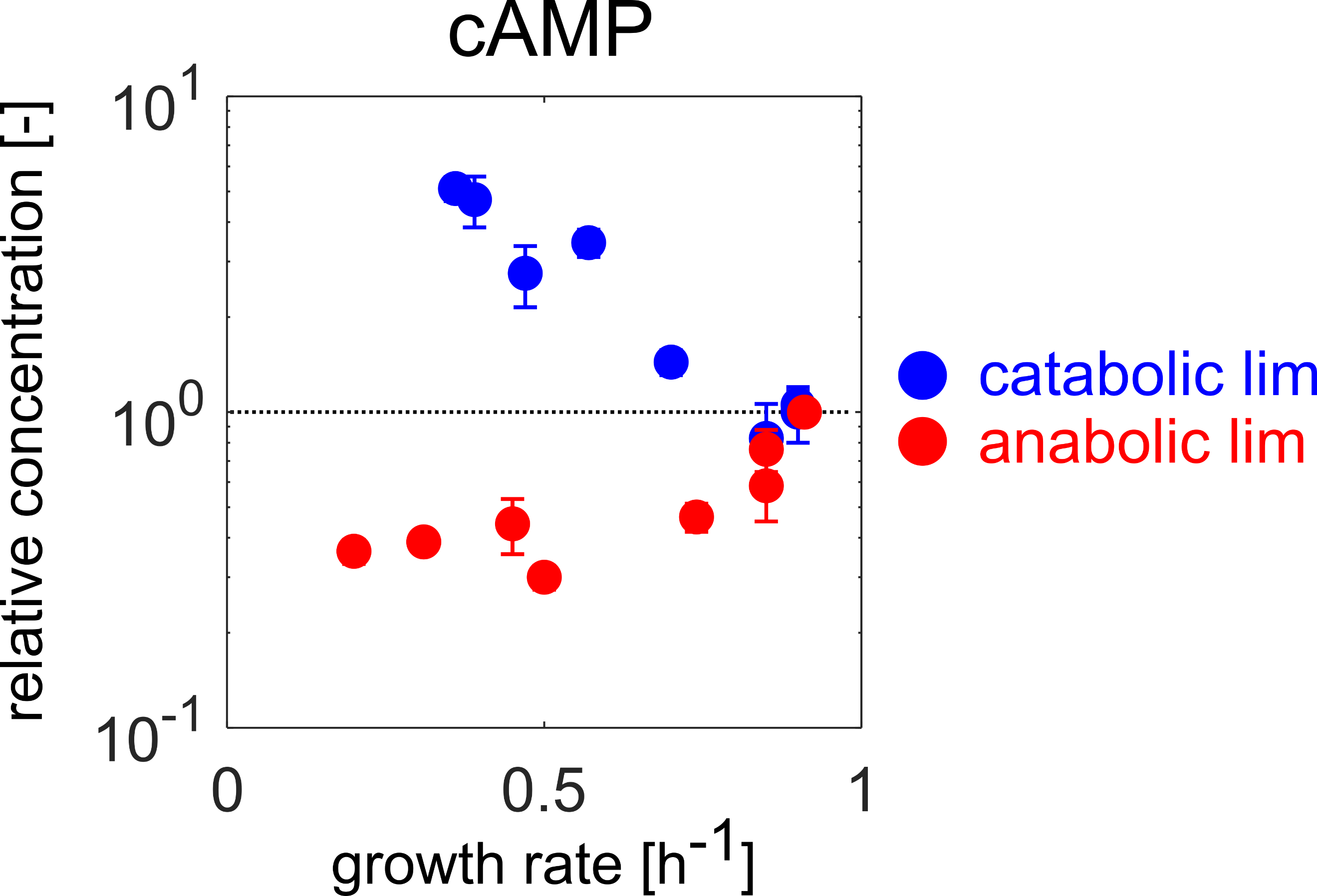


**Appendix figure S2. Cyclic AMP concentration indicates differential activity of Crp in catabolic and anabolic limitation.** Intracellular concentration of cyclic AMP, the small molecule activator of Crp, in catabolic (blue) and anabolic (red) limitation (relative to NCM3722 WT) as determined by LC-MS/MS. Data shown are the mean of three biological replicates.

**
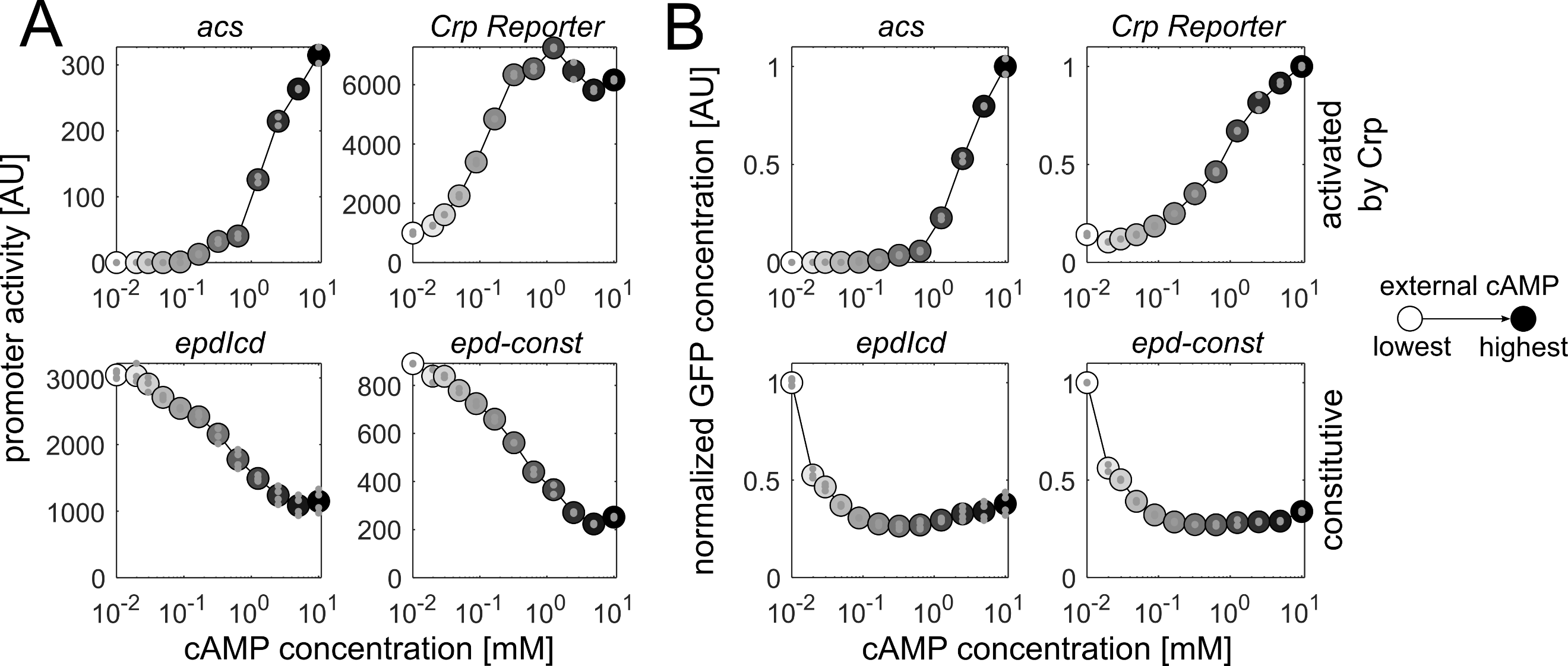
**

**Appendix figure S3. Steady state promoter activity (A) and GFP concentration (B) of Crp activated and constitutive transcriptional fluorescent reporters in NQ1399 at varying external cyclic AMP concentrations.** Transcriptional reporters expressing GFP were obtained from (Zaslaver *et al*, 2006; Gerosa *et al*, 2013; Kochanowski *et al*, 2017) and transferred to NQ1399 by heat-shock transformation. Cultivation was performed as described in the main text, and steady state promoter activity (quantified as OD-normalized rate of GFP synthesis) during the 1.5h window of maximal exponential growth was quantified as described previously (Gerosa *et al*, 2013; Kochanowski *et al*, 2013). External cyclic AMP concentrations from lowest to highest: 0.01, 0.02, 0.03, 0.05, 0.09, 0.17, 0.32, 0.64, 1.26, 2.51, 5.01, 10.01 mM. Top row: two promoters that are activated by Crp. Promoter ‘*Crp Reporter*’ was constructed from the epd promoter by removing the additional Cra binding site, as described previously (Kochanowski *et al*, 2017). Second row: synthetic constitutive promoters (Gerosa *et al*, 2013). Steady state GFP concentrations were directly calculated from promoter activity data depicted as described previously (Gerosa *et al*, 2013) (GFP concentration = promoter activity/growth rate), and normalized to the maximal value for each promoter. Small grey circles: individual biological replicates (n = 2-4). Large circles: mean across replicates..

**
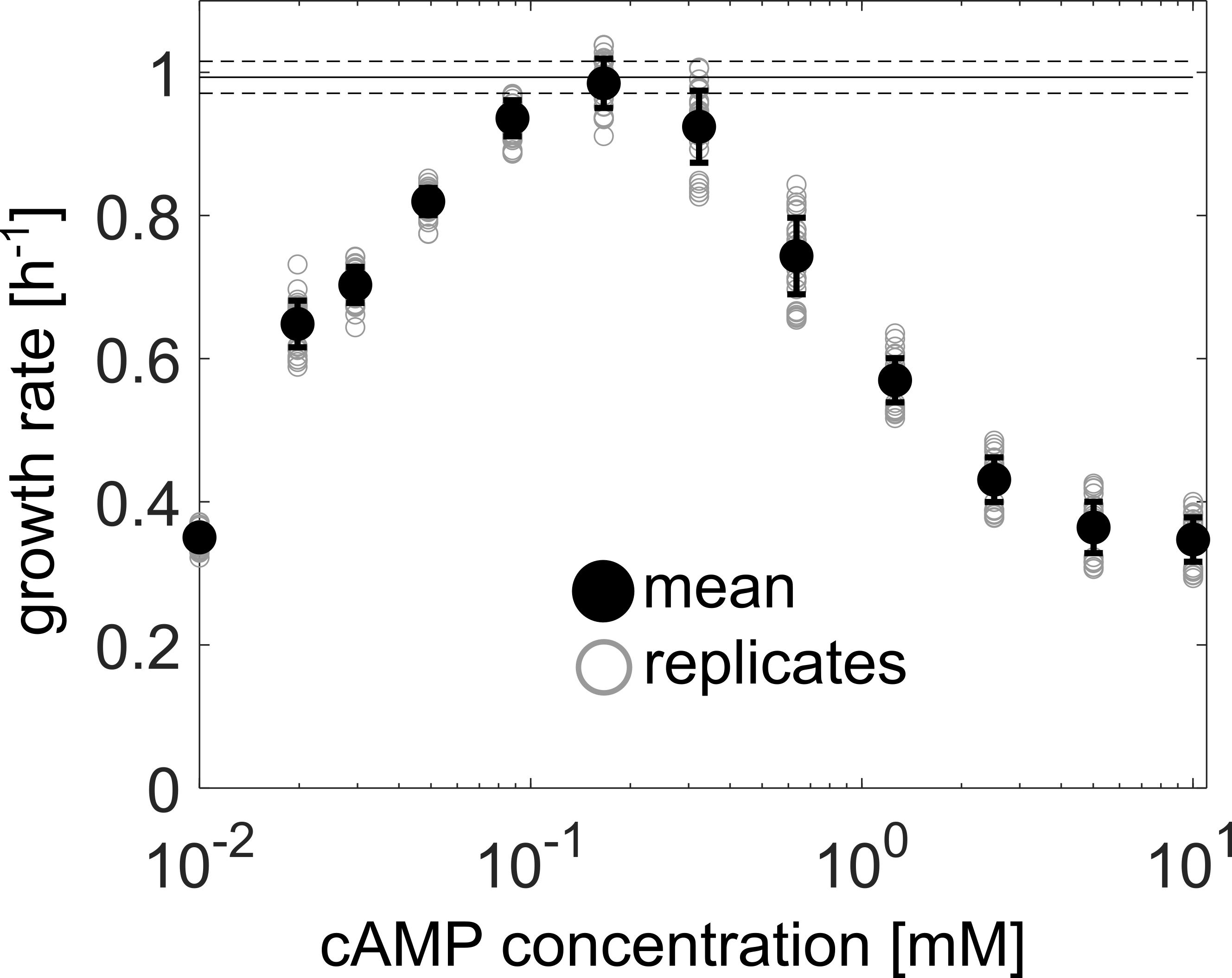
**

**Appendix Figure S4. Growth rate of NQ1399 (no internal cyclic AMP production) in M9 glucose (2g/L) supplemented with varying external cyclic AMP concentrations.** Cultivation of NQ1399 was performed in an automated 96-well plate reader as described in the main text. Culture growth rates for each culture were calculated during the 1.5h window in which the cultures exhibited the maximal growth rate. Black circles: mean growth rate across 32 biological replicates (across four independent experiments). Error bars denote standard deviation. Grey circles: growth rate of each replicate. Black horizontal line: growth rate of wild-type cells (NCM3722) in M9 glucose media (without added cAMP). Dashed lines: corresponding standard deviation (n = 4).


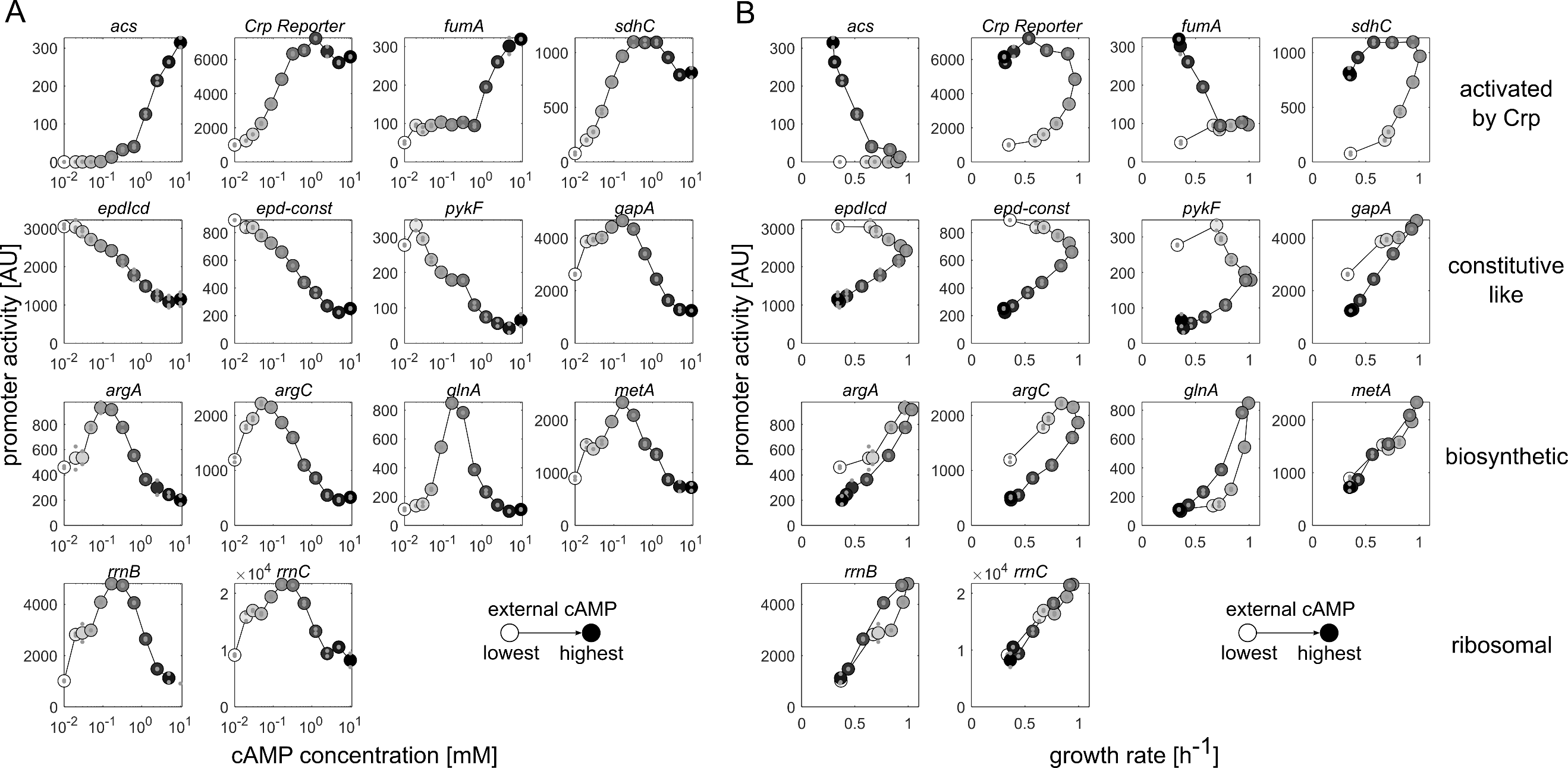


**Appendix Figure S5. Steady state promoter activity of different transcriptional fluorescent reporters in NQ1399 at varying external cyclic AMP concentrations.** Transcriptional reporters expressing GFP were obtained from (Zaslaver *et al*, 2006; Gerosa *et al*, 2013, 2015; Kochanowski *et al*, 2017) and transferred to NQ1399 by heat-shock transformation. Cultivation was performed as described in the main text, and steady state promoter activities - defined as OD-normalized GFP production rates – during the 1.5h window of maximal exponential growth were quantified as described previously (Gerosa *et al*, 2013; Kochanowski *et al*, 2013). External cyclic AMP concentrations from lowest to highest: 0.01, 0.02, 0.03, 0.05, 0.09, 0.17, 0.32, 0.64, 1.26, 2.51, 5.01, 10.01 mM. Top row: four promoters that are activated by Crp (see Ref. (Kochanowski *et al*, 2017) for details). Promoter ‘*Crp Reporter*’ was constructed by putting the Crp binding site of the epd promoter upstream of the synthetic constitutive promoter *epdIcd* (Gerosa *et al*, 2013, 2015). Second row: constitutive-like promoters (that is, promoters that were previously found to largely behave like synthetic constitutive promoters across multiple conditions (Gerosa *et al*, 2013, 2015; Kochanowski *et al*, 2017)). Third row: four promoters of proteins that are part of amino acid biosynthesis pathways. Bottom row: ribosomal RNA promoters *rrnB* and *rrnC*. Small grey circles: individual biological replicates (n = 2-4). Large circles: mean across replicates. **A)** Data plotted against external cAMP concentration. **B)** Same data plotted against respective growth rate. Note: data for acs, Crp Reporter, epdIcd, epd-const promoters are the same as shown in Figure S3.

**
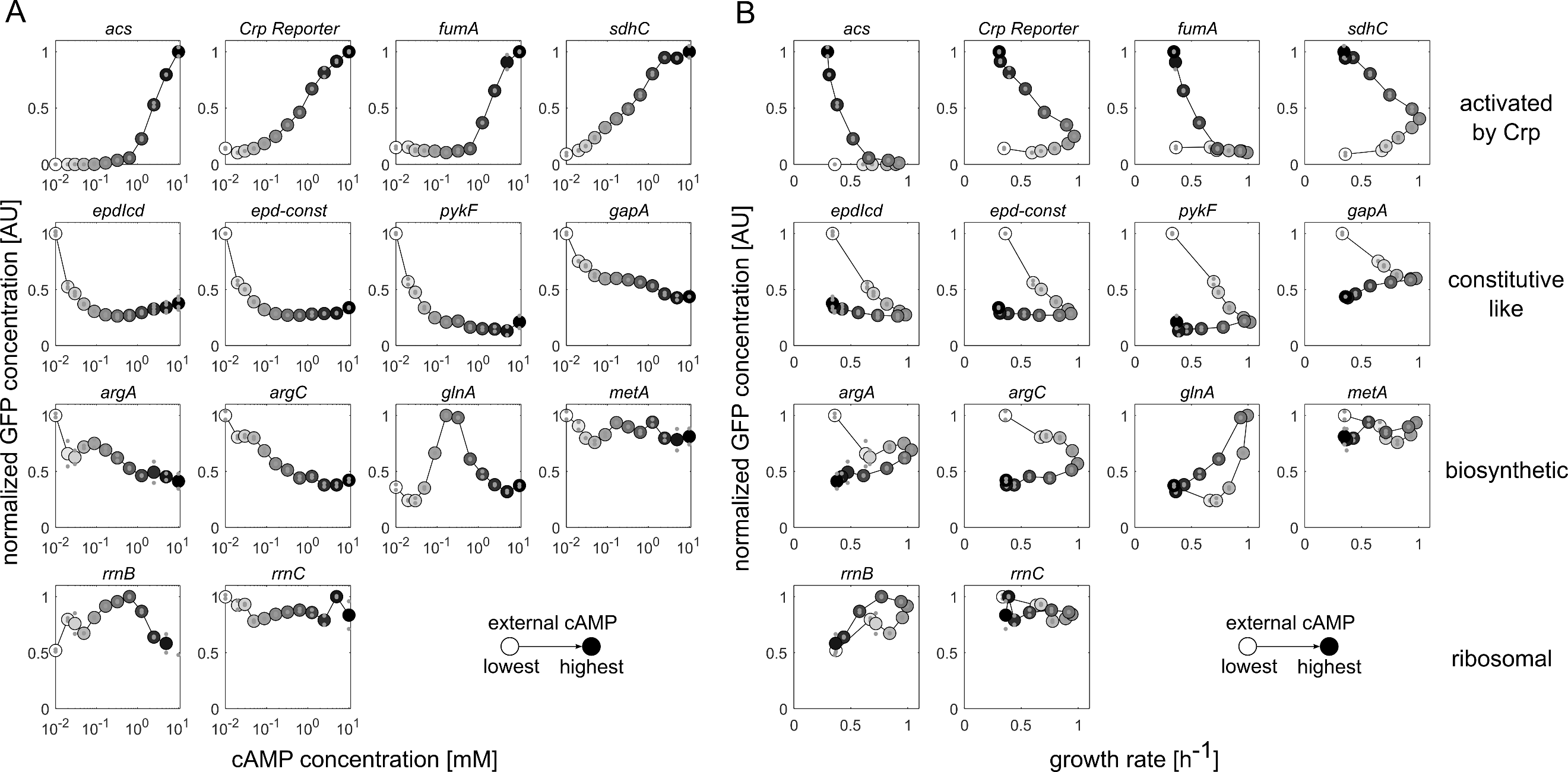
**

**Appendix Figure S6. Steady state GFP concentration of different transcriptional fluorescent reporters in NQ1399 at varying external cyclic AMP concentrations.** Steady state GFP concentrations were calculated from promoter activity data depicted in Appendix Figure S5 as described previously (Gerosa *et al*, 2013): GFP concentration = promoter activity/growth rate. Subsequently, data were normalized (for each promoter separately) to the maximal GFP concentration across conditions. Small grey circles: individual biological replicates (n = 2-4). Large circles: mean across replicates. **A)** Data plotted against external cAMP concentration. **B)** Same data plotted against respective growth rate. Note: data for acs, Crp Reporter, epdIcd, epd-const promoters are the same as shown in Figure S3.

**
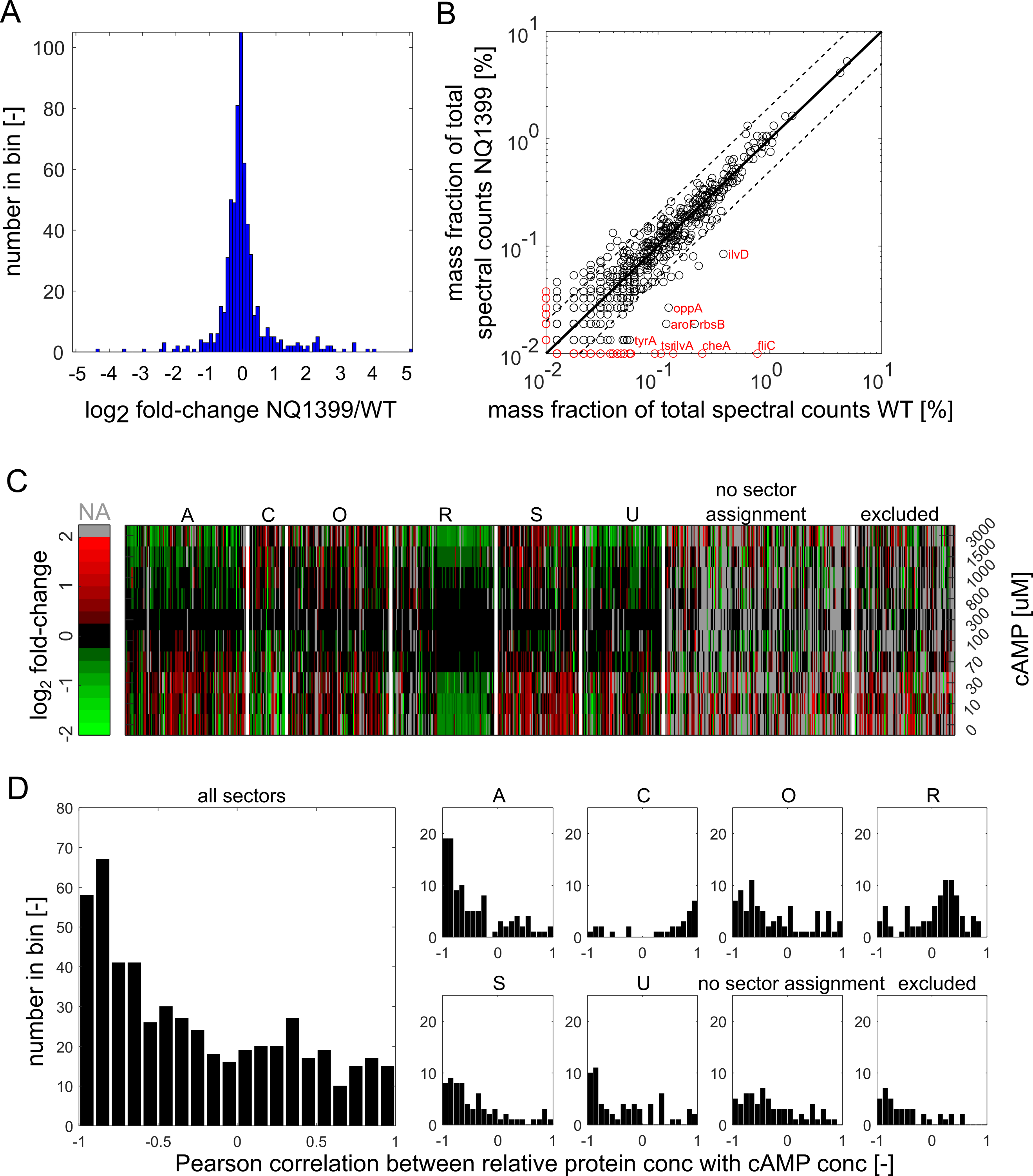
**

**Appendix Figure S7. Impact of external cAMP on NQ1399 proteome.** **A-B)** **Comparison of the proteome of WT and NQ1399 at growth-matched conditions (i.e. with 0.2 mM cAMP).** **A)** Histogram of protein ratios as determined by relative protein quantification. 89% of the detected proteins had log_2_ fold-changes between -1 and 1. **B)** Fraction of spectral counts in WT (x-axis) and NQ1399 (y-axis) for all detected proteins, in percent. Red circles: proteins that were only detected in one strain (name tags are added if these proteins account for more than 0.1% of the total spectral counts). Black circles with name tags: proteins whose spectral counts deviate more than 4-fold (log2 fold-changes below -2 or above 2) between WT and NQ1399. To aid visualization, spectral counts below 0.01% were set to 0.01%. Only proteins, which were quantified in both replicates in at least one of the strains, were considered. Data shown are the geometric mean of two replicates. **C-D) Relative protein concentrations in NQ1399 at varying external cAMP concentrations.** **C)** Heatmap of relative protein concentrations in NQ1399 (normalized to reference condition 0.3 mM cAMP). Individual proteins were sorted according to sector assignment as in (Hui *et al*, 2015) (e.g. A: anabolic proteins, C: catabolic proteins, R: ribosomal proteins). Proteins without sector assignment in the original study, and proteins not detected in the control experiments depicted in A) and B), were labeled as “no sector assignment”. Proteins that were detected as outliers when comparing WT and NQ1399 with 0.2 mM cyclic AMP (see Appendix Figure S7A-B) were labeled as “excluded”. Grey: protein not detected in the respective condition. **D)** Distribution of Pearson correlation coefficients between log2 of cAMP concentration and log2 of respective relative protein concentration. Large plot: all proteins detected in study with data in at least 80% of conditions. Subplots: proteins separated by sector assignment as described in C).


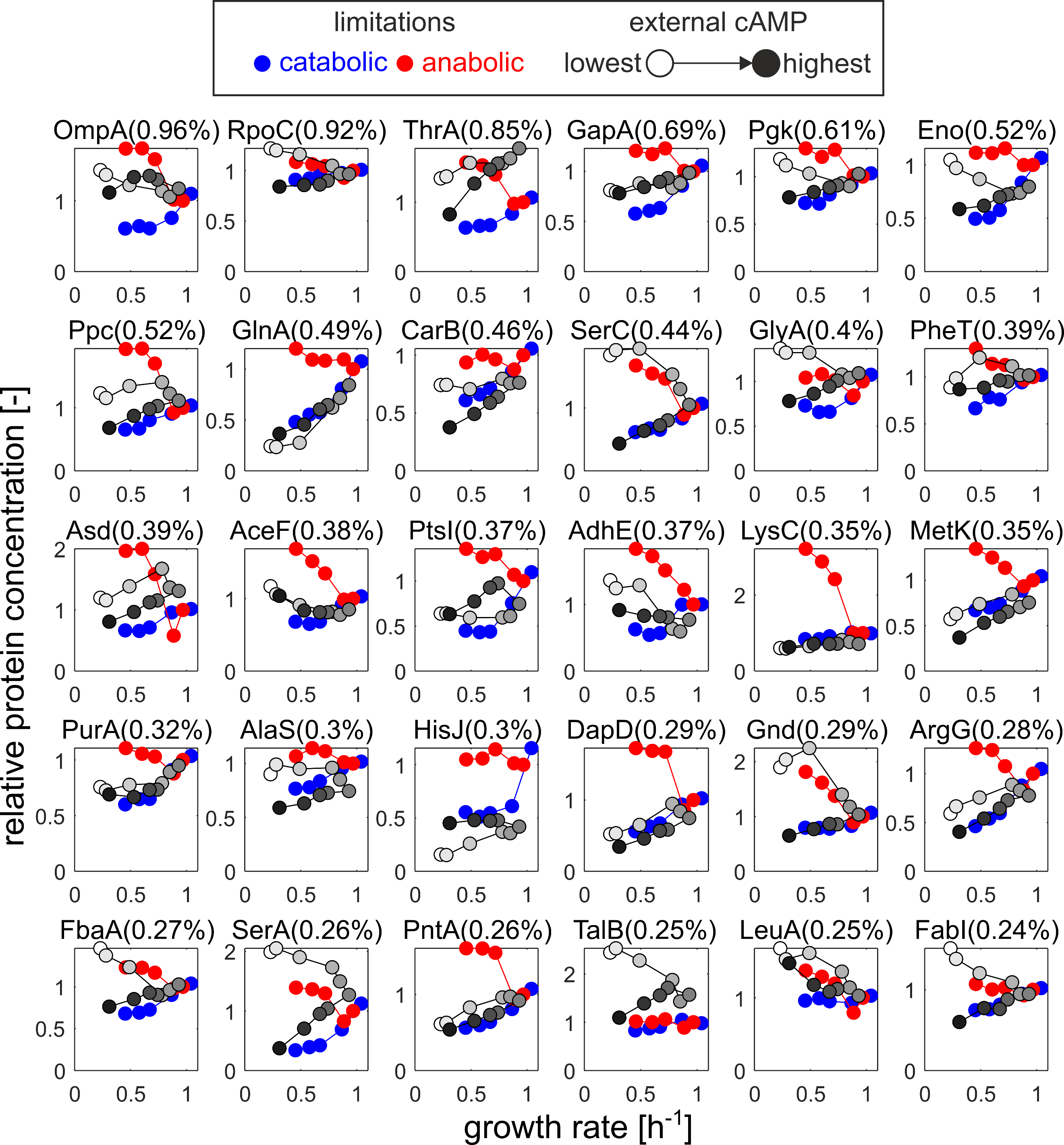


**Appendix Figure S8. Relative concentration of 30 most abundant anabolic proteins.** Data for catabolic and anabolic limitations for “A-sector proteins” from (Hui *et al*, 2015) are shown as blue and red circles. For the cAMP titration data set, protein concentrations were first normalized to the cAMP concentration yielding the maximal growth rate, and then further adjusted to account for differences between strain NQ1399 and WT (using the data depicted in Appendix Figure S7A). Number in brackets: mean protein abundance (as percentage of total detected proteome) across all limitations and cAMP titration data.


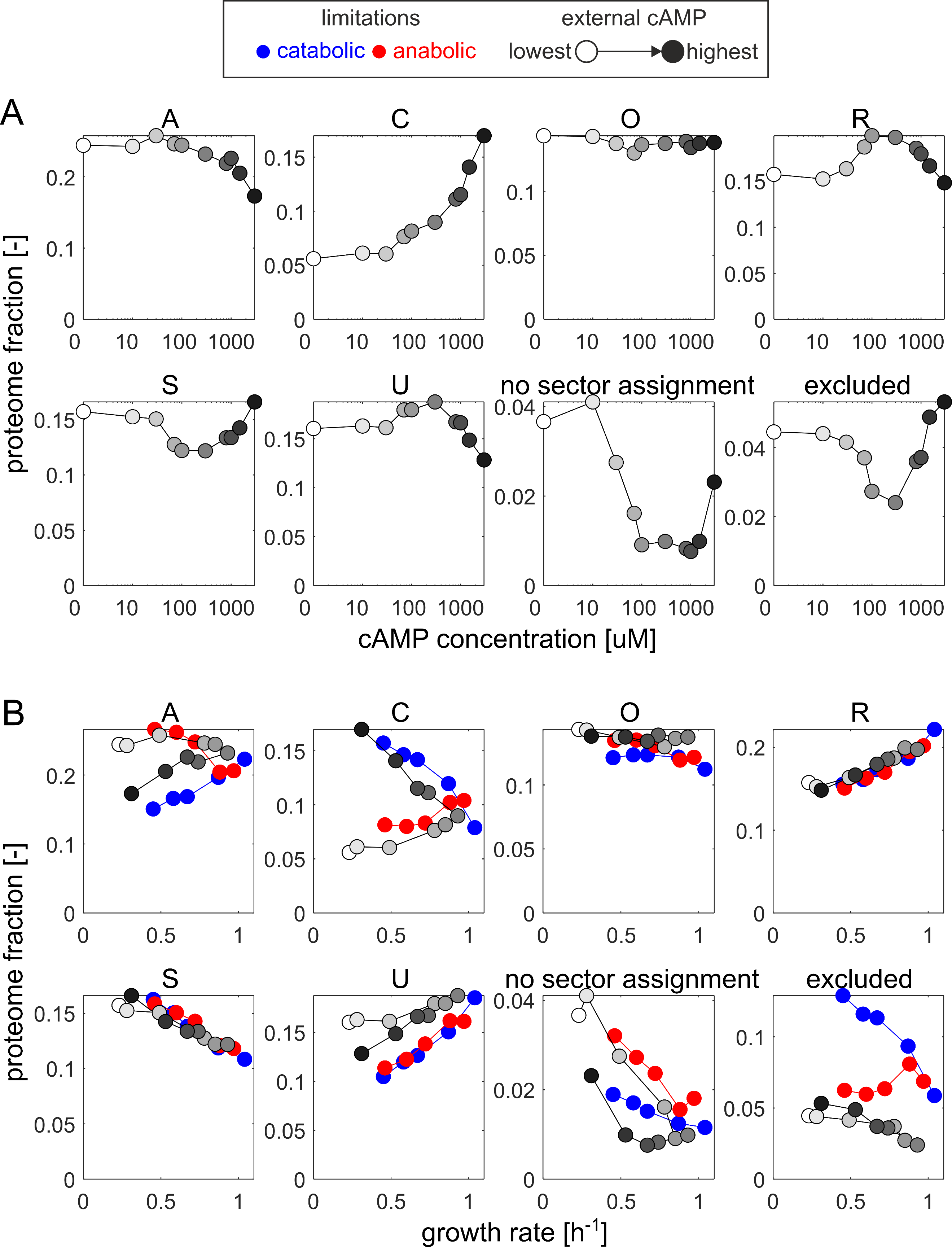


**Appendix Figure S9. Proteome sectors in cAMP titration and catabolic/anabolic limitations.** Data show fractions of total (detected) proteome. Sector assignments of proteins as in (Hui *et al*, 2015) (i.e., A: anabolic proteins, C: catabolic proteins, R: ribosomal proteins). Data for catabolic and anabolic limitation from this publication was re-analyzed to account for additional proteins that were detected in the cAMP titration experiments. Proteins without sector assignment in the original study were labeled as “no sector assignment”. Proteins that were detected as outliers when comparing WT and NQ1399 with 0.2 mM cyclic AMP (see Appendix Figure S7) were labeled as “excluded”. **A)** cAMP titration data plotted against cAMP concentration. **B)** Same data plotted against respective growth rate and overlaid with catabolic (blue) and anabolic (red) limitation data. Note: data for A and C sectors are the same as shown in Figure 1C.


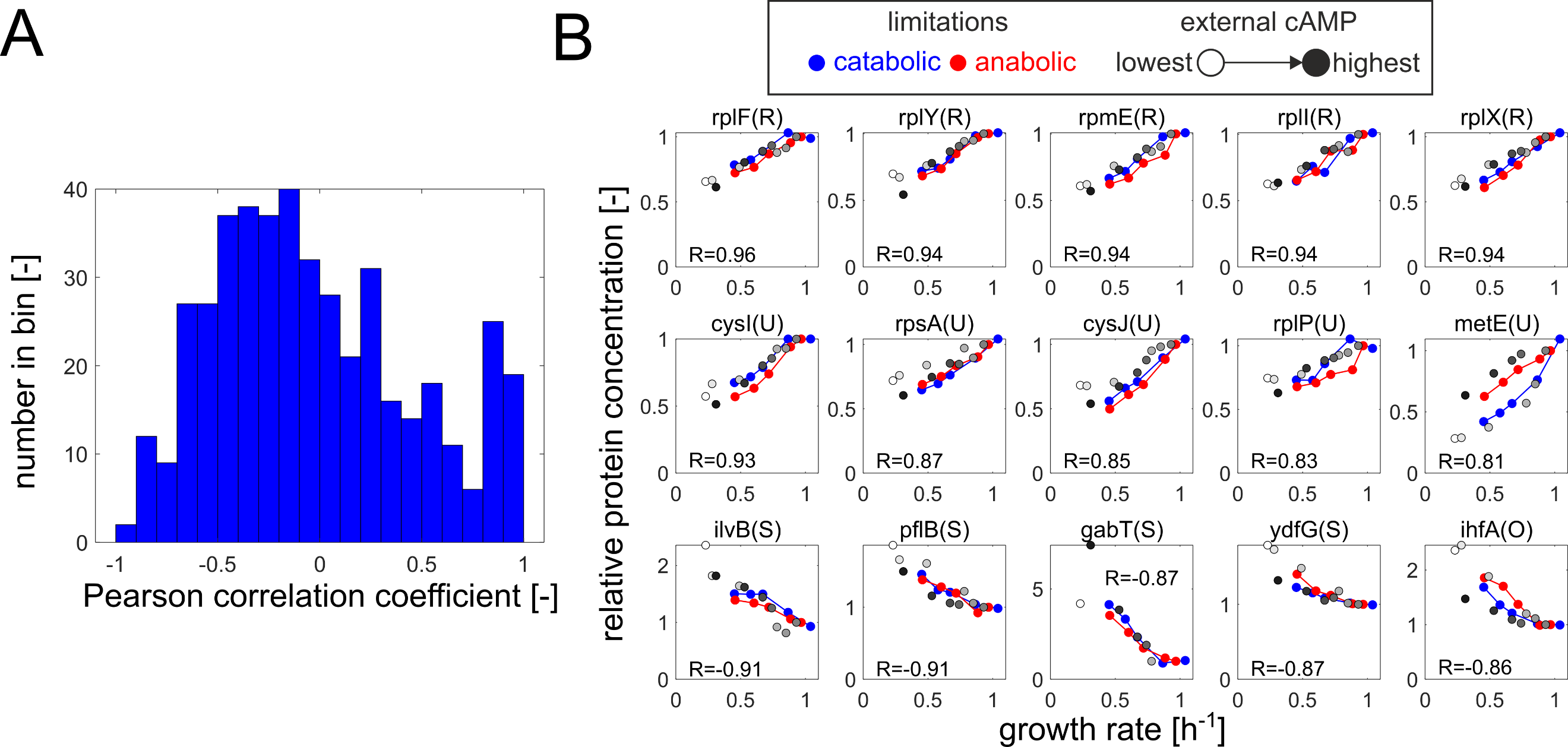


**Appendix Figure S10. Identifying proteins which maintain a strict correlation with growth rate in both limitations and cyclic AMP titration. A)** Histogram of Pearson correlation coefficients between relative protein concentration and growth rate. Data for carbon and anabolic limitation, as well as cyclic AMP titration, were merged to calculate the overall correlation coefficient. Only proteins which were detected in at least 80% of the samples were considered. **B)** Top row: proteins assigned to sector “R” (ribosomal sector) with the highest positive correlation coefficients. Middle row: proteins not assigned to sector “R” (sector assignment in brackets) with the highest positive correlation coefficients. Bottom row: proteins with the strongest negative correlation between relative protein concentration and growth rate.


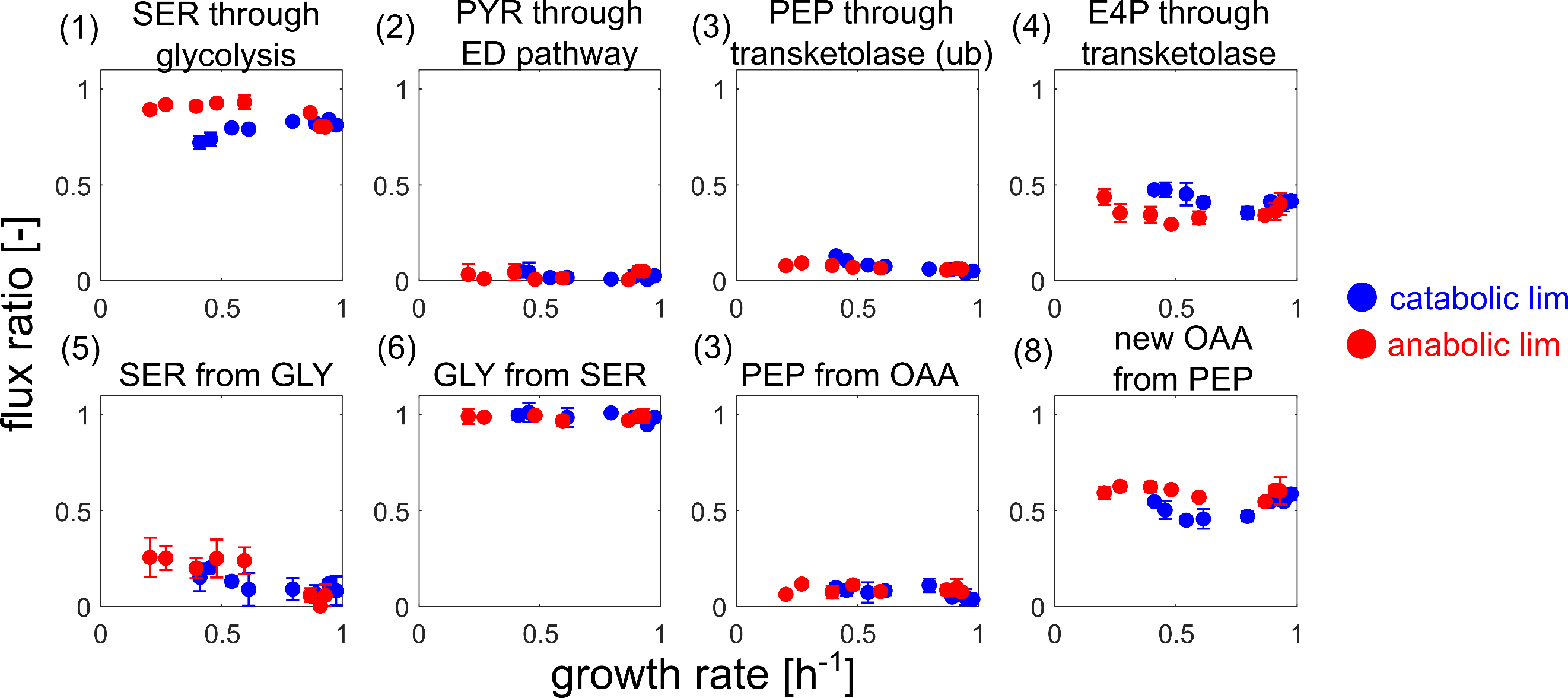


**Appendix Figure S11. Flux ratios in catabolic (blue) and anabolic (red) limitation as determined by ^13^C flux analysis.** (1) Ratio of glycolysis to sum of Pentose phosphate (PP) + Entner Doudoroff (ED) pathway. (2) Flux ratio of pyruvate (PYR) derived through ED pathway. (3) Upper bound of phosphoenolpyruvate (PEP) derived through transketolase. (4) Upper bound of E4P derived through transketolase. (5) Flux ratio of serine (SER) derived from glycine (GLY) through glycine cleavage pathway. (6) Flux ratio of glycine derived from serine. (7) PEP derived from oxaloacetate (OAA) through PEP carboxykinase (Pck). (8) OAA derived from PEP through PEP carboxylase (Ppc). Error bars denote standard deviation of three biological replicates. Not shown: flux ratios for malic enzymes and glyoxylate shunt, which were found to be zero in all tested conditions.


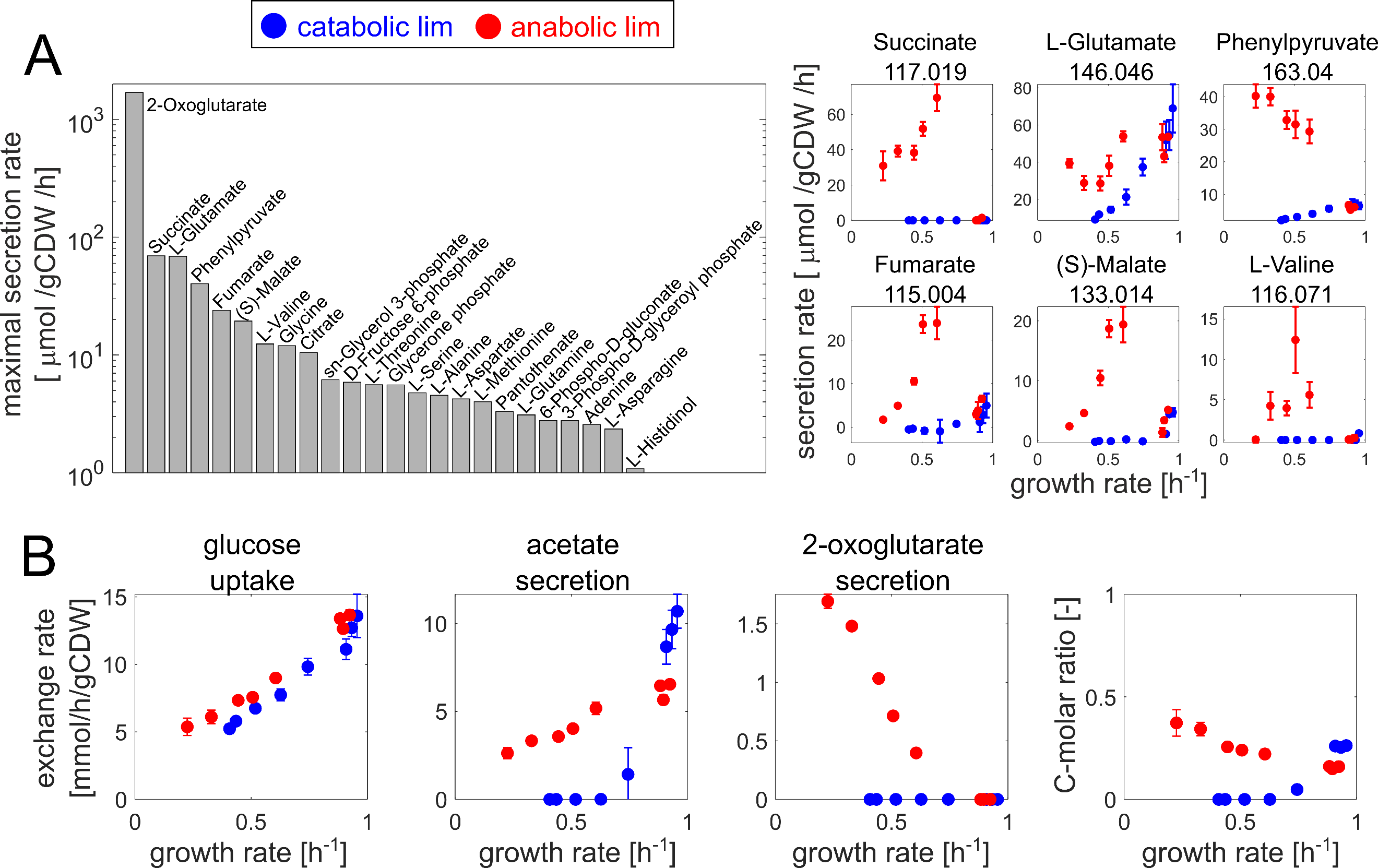


**Appendix Figure S12. Metabolite exchange rates in carbon (blue) and anabolic (red) limitation. A)** Absolute secretion rates as determined by flow-injection time-of-flight (FIA-TOF) mass spectrometry (Fuhrer *et al*, 2011). Left: maximal secretion rates across all conditions. Right: secretion rates of six minor secretion products plotted against the growth rate in the corresponding conditions. Numbers underneath metabolite names denote m/z. Secretion rates were calculated as described in the methods section. Error bars denote standard deviation of three biological replicates. **B)** Rates of major exchange products plotted against the growth rate in the corresponding conditions. Glucose and acetate exchange rates were calculated based on the quantification of extracellular glucose and acetate by colorimetric enzyme assays. 2-oxoglutarate secretion rate was quantified by FIA-TOF mass spectrometry as described above. Right-most panel: relative secretion rate calculated as the carbon-normalized fraction of glucose that is being secreted by acetate or 2-oxoglutarate. Error bars denote standard deviation of three biological replicates. Note: data for glucose uptake and acetate/2-oxoglutarate secretion rates are the same as shown in Figure 2A.

**
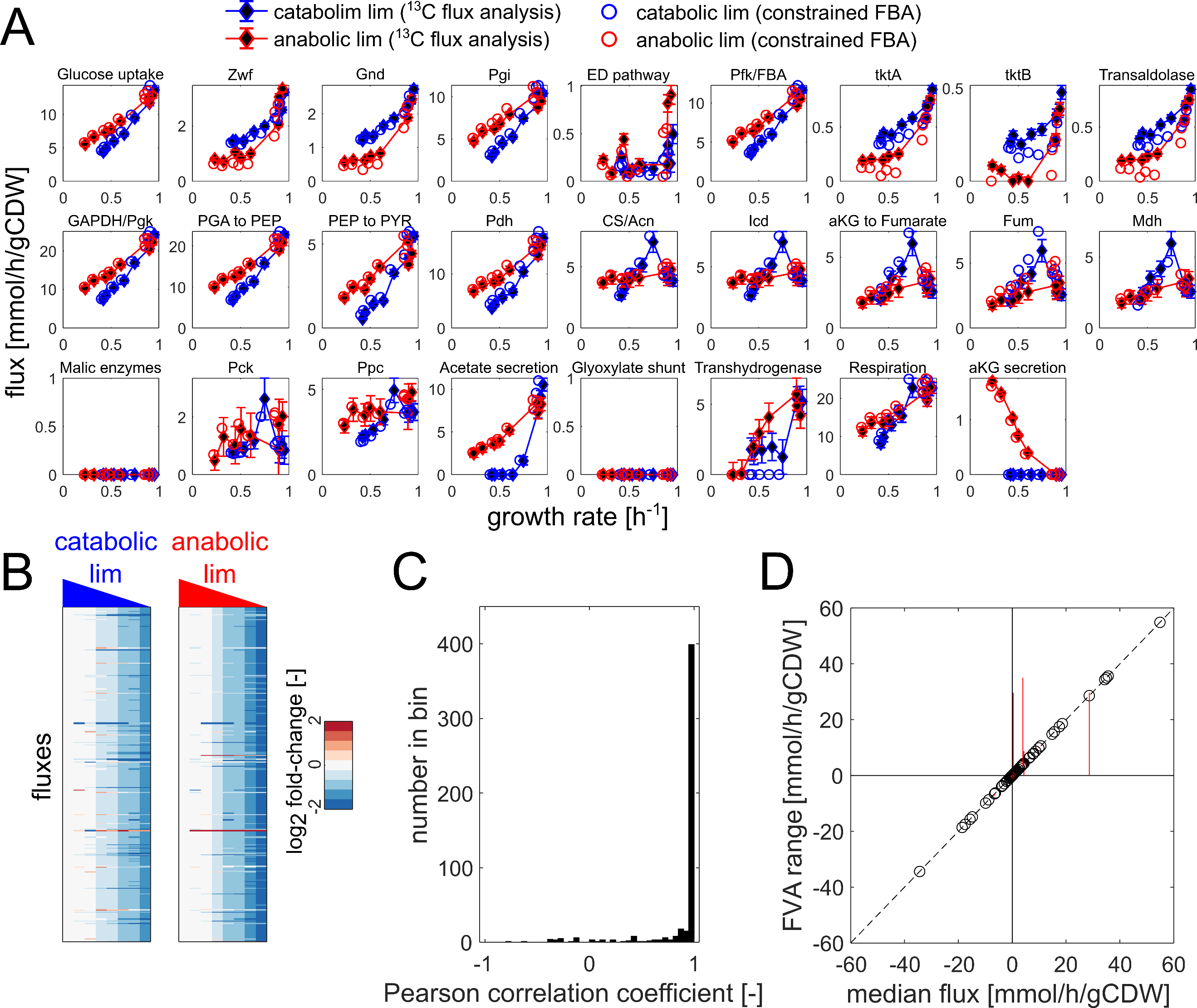
**

**Appendix Figure S13. Metabolic fluxes in catabolic and anabolic limitation as determined by ^13^C-flux analysis and constrained flux balance analysis (FBA). A)** Comparison of central metabolic fluxes as determined by ^13^C-flux analysis and FBA. Blue: catabolic limitation. Red: anabolic limitation. Pgi: Phosphoglucoseisomerase. Pfk/FBA: flux from F6P to 2 GAP. Gapdh/Pgk: flux from GAP to 3PG. Pgm/Eno: flux from 3PG to PEP. PEP to PYR: total flux from PEP to PYR (PTS + PYR kinase). Zwf: flux from G6P to 6PG. Gnd: flux from 6PG to P5P. Pdh: flux from PYR to acetyl-CoA. CS/Acn: flux from OAA + acetyl-CoA to isocitrate. Icd: flux through isocitrate dehydrogenase. aKG to Succinate: flux through aKG dehydrogenase, succinyl-CoA synthethase and succinate oxidoreductase. Fum: flux through fumarase. Mdh: flux through Malate dehydrogenase. Pck: flux through PEP carboxykinase. Ppc: flux through PEP carboxylase. Respiration: uptake rate of O_2_. Error bars denote standard deviation of flux estimate based on error propagation of the standard deviations of flux ratios and exchange rates (three biological replicates). **B)** Overview of FBA estimates of metabolic fluxes in catabolic and anabolic limitations. Data shown: relative to NCM3722 wildtype in M9 glucose and log2-normalized. To simplify visualization, only reactions that carry flux in all tested conditions are shown. **C)** Distribution of Pearson correlation coefficients between fluxes and respective growth rate across conditions. Only reactions that carry flux in at least 50% of tested conditions were considered. **D)** Flux variability analysis (FVA) of flux estimates. Circles: median flux for each reaction with non-zero flux in at least one condition. Red vertical lines: median of respective minimal and maximal FVA range.

**
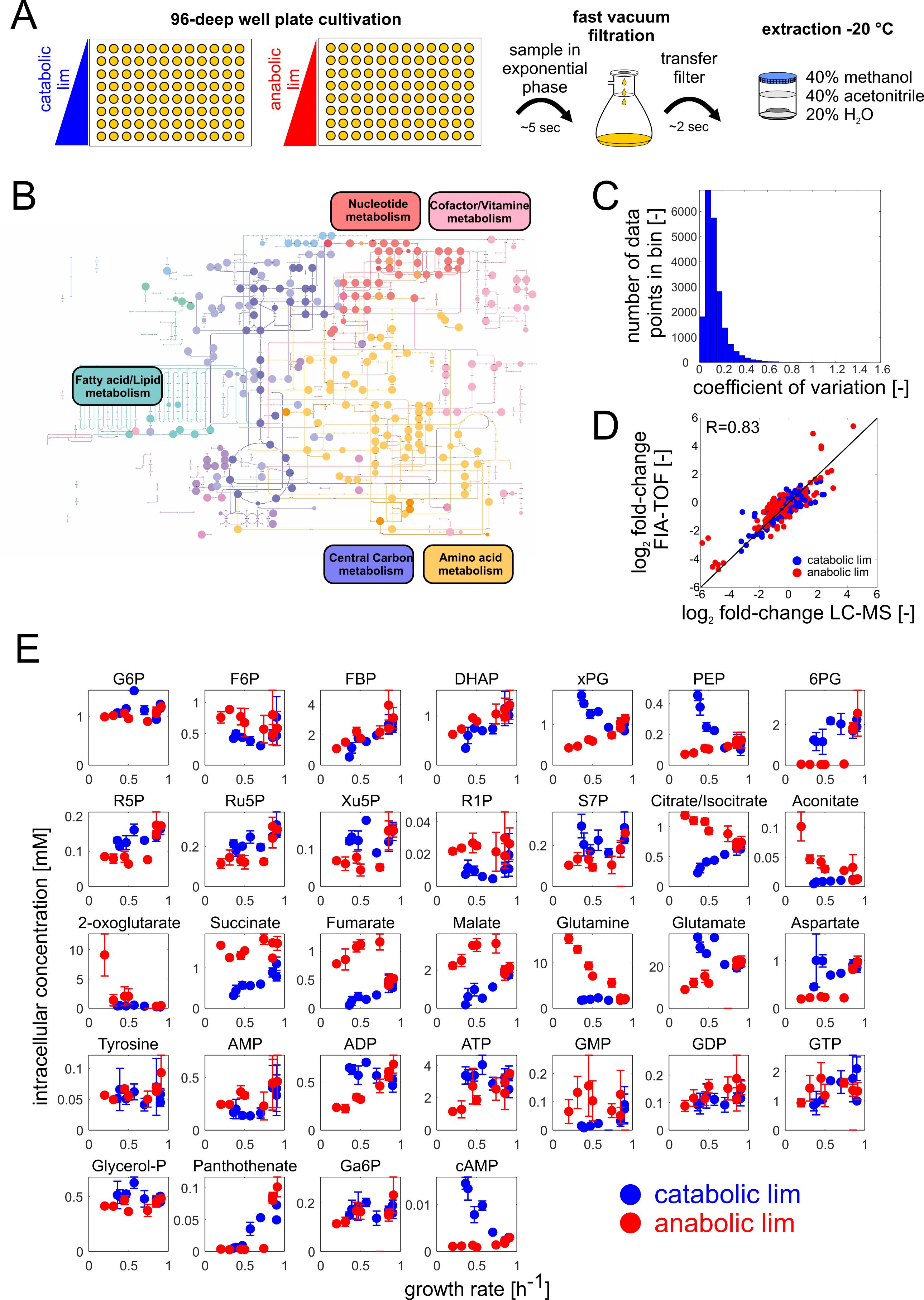
**

**Appendix Figure S14. Intracellular metabolome response in catabolic (blue) and anabolic (red) limitation. A)** Schematic of experimental setup to determine the intracellular metabolome of carbon and anabolic limitation strains. Cultures were grown in 96-deep well plates (fill volume 1.2 mL) as described previously (Kochanowski *et al*, 2017). During mid-exponential growth (OD 0.5 – 0.7), cells from 1 mL culture samples were harvested by fast filtration and quenched/extracted with cold methanol extraction (Link *et al*, 2012, 2013). Samples for untargeted and targeted metabolomics were taken in parallel from cultures growing in the same 96-well plate. **B)** KEGG metabolic map of *E.coli*. Filled circles denote metabolites that were quantified by untargeted metabolomics (flow-injection time-of-flight (FIA-TOF) mass spectrometry, annotation with 0.001 Da mass accuracy of deprotonated ions based on KEGG *E. coli* model). In total, 430 unique ions were annotated, mapping to 900 unique metabolites. **C)** Distribution of relative standard deviations (three biological replicates, two technical replicates per biological replicate) across all ions and conditions. The median coefficient of variation is 14%. **D)** Comparison of log2 fold-changes (relative to NCM3722 wildtype in M9 glucose) for all metabolites that were quantified by both untargeted (FIA-TOF) and targeted (LC-MS/MS) metabolomics. R: Pearson correlation coefficient. **E)** Absolute intracellular concentration of central carbon metabolites as determined by LC-MS/MS. Error bars denote standard deviation of three biological replicates. In all conditions, the energy charge (defined as [ATP + 0.5 ADP]/[ATP + ADP + AMP]) was between 0.78 and 0.9, suggesting a stable physiological state. Note: cAMP data are the same as shown in Figure S2.


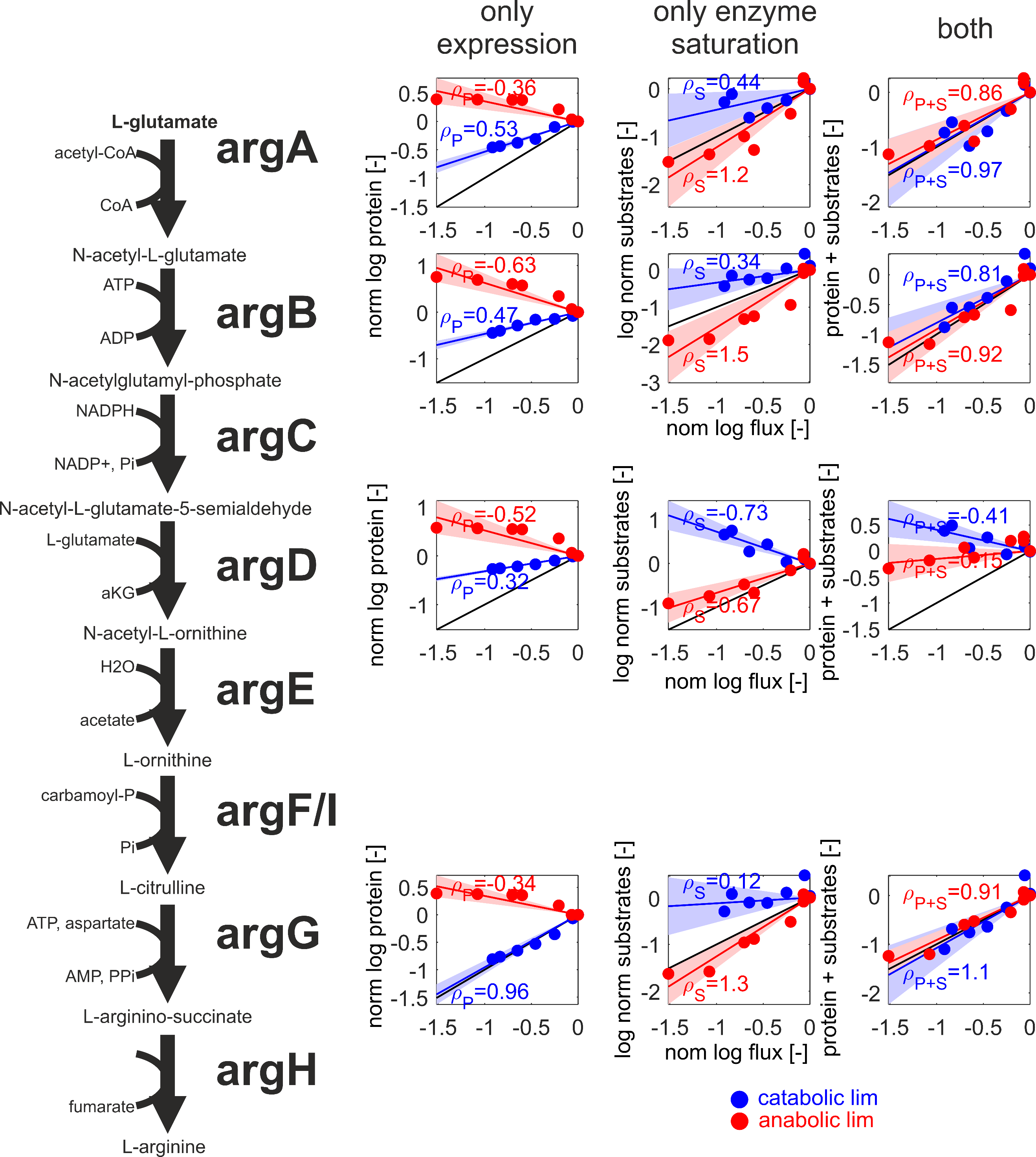


**Appendix Figure S15. Regulation analysis of arginine biosynthesis pathway in catabolic (blue) and anabolic (red) limitation.** Left: schematic depiction of arginine biosynthesis pathway including all substrates and cofactors. In bold next to each reaction are the associated proteins. Right: relationship between observed flux changes and changes in protein (left column) or substrate concentration (middle column), as well as their combined effect (right column). Blue and red lines denote the estimated regulation coefficients $\rho_{P}$ and $\rho_{S}$ (calculated by linear regression) between measured flux and protein concentrations for carbon and anabolic limitation, respectively. Shaded areas denote the standard error of each estimate. Black lines denote full proportionality. Note: data for ArgB are the same as shown in Figure 3C.


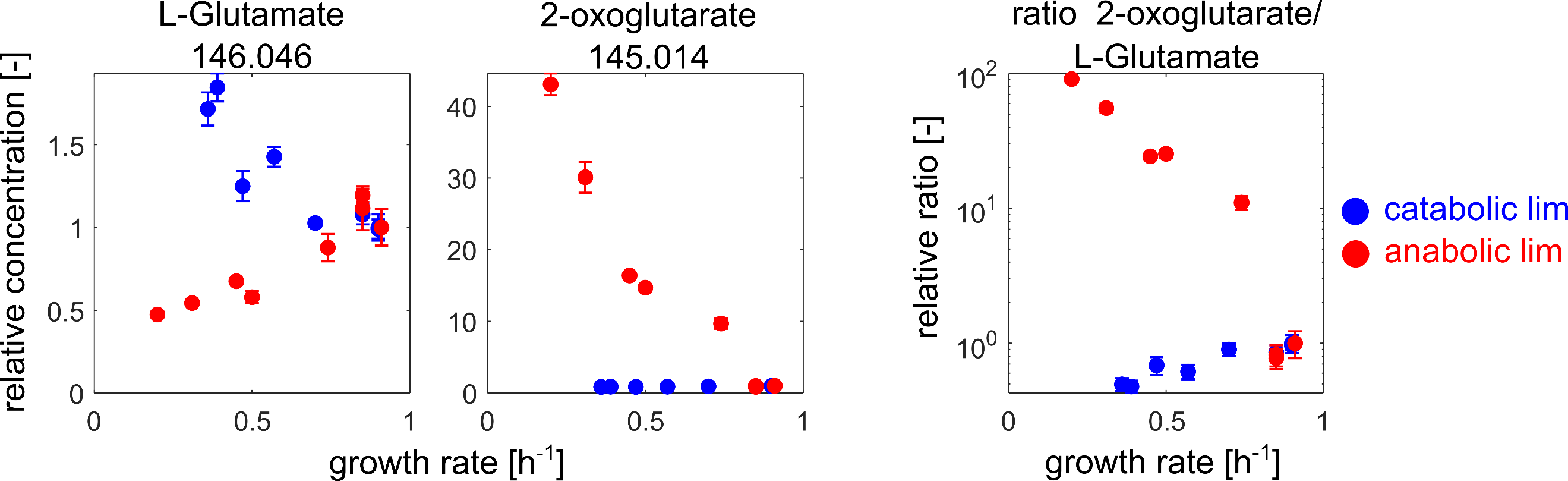


**Appendix Figure S16.** Left and middle panel: Relative intracellular concentration of L-Glutamate and 2-oxoglutarate (data shown are the same as in main figure 3). Right panel: Ratio of relative 2-oxoglutarate and L-glutamate concentration. An increase in this ratio reduces the driving force of glutamate dependent trans-aminases, for which L-Glutamate is a substrate and 2-oxoglutarate is a product. Note: data for L-glutamate and 2-oxoglutarate are the same as in Figure 3A.


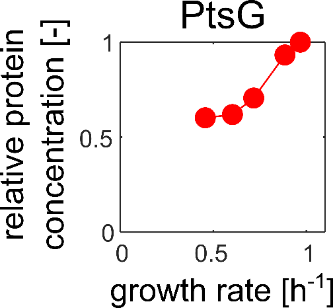


**Appendix Figure S17.** Relative PtsG protein concentration in anabolic limitation. Data from (Hui *et al*, 2015).


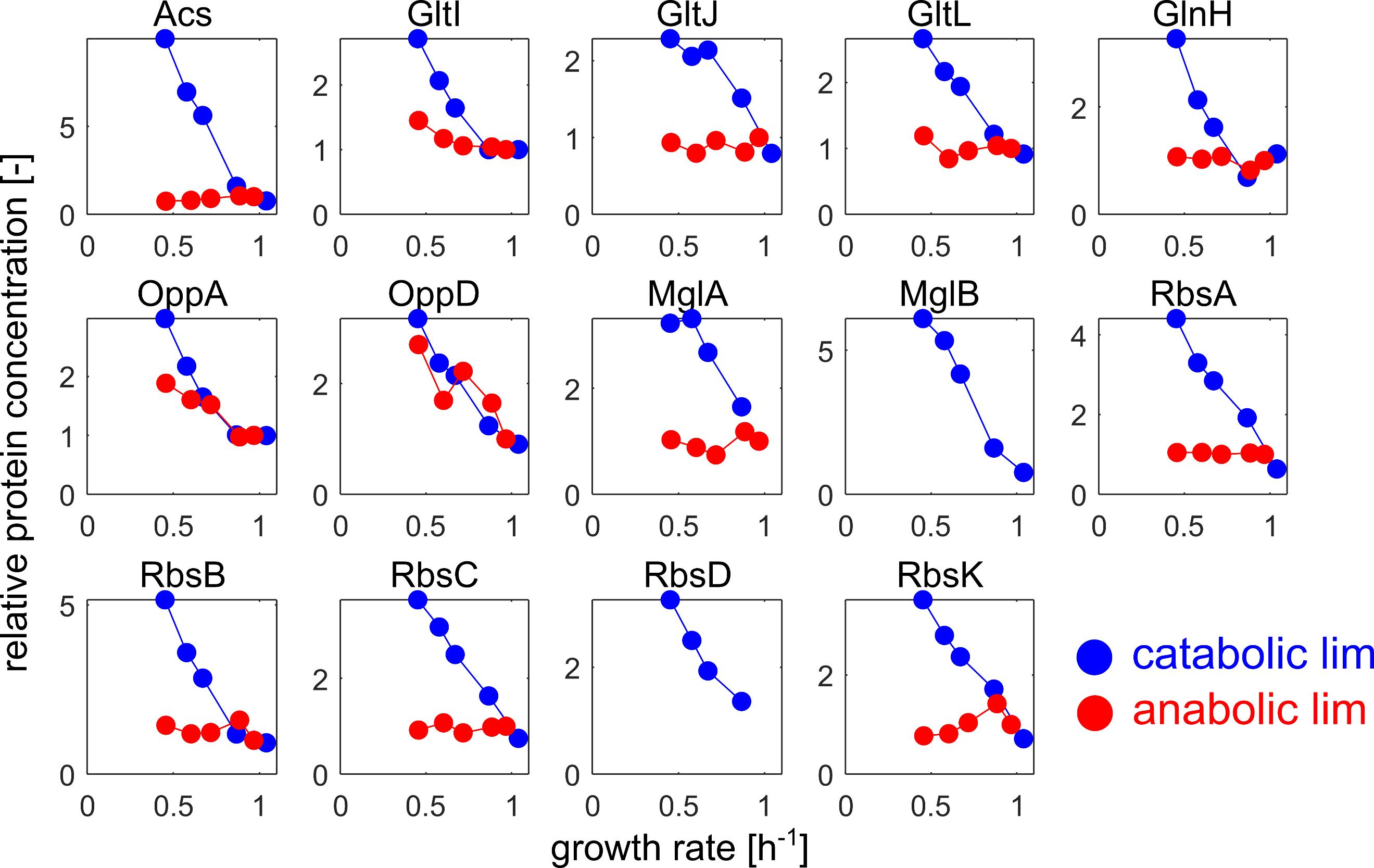


**Appendix Figure S18.** Relative concentration of selected transporter proteins in catabolic and anabolic limitations. Data from (Hui *et al*, 2015).


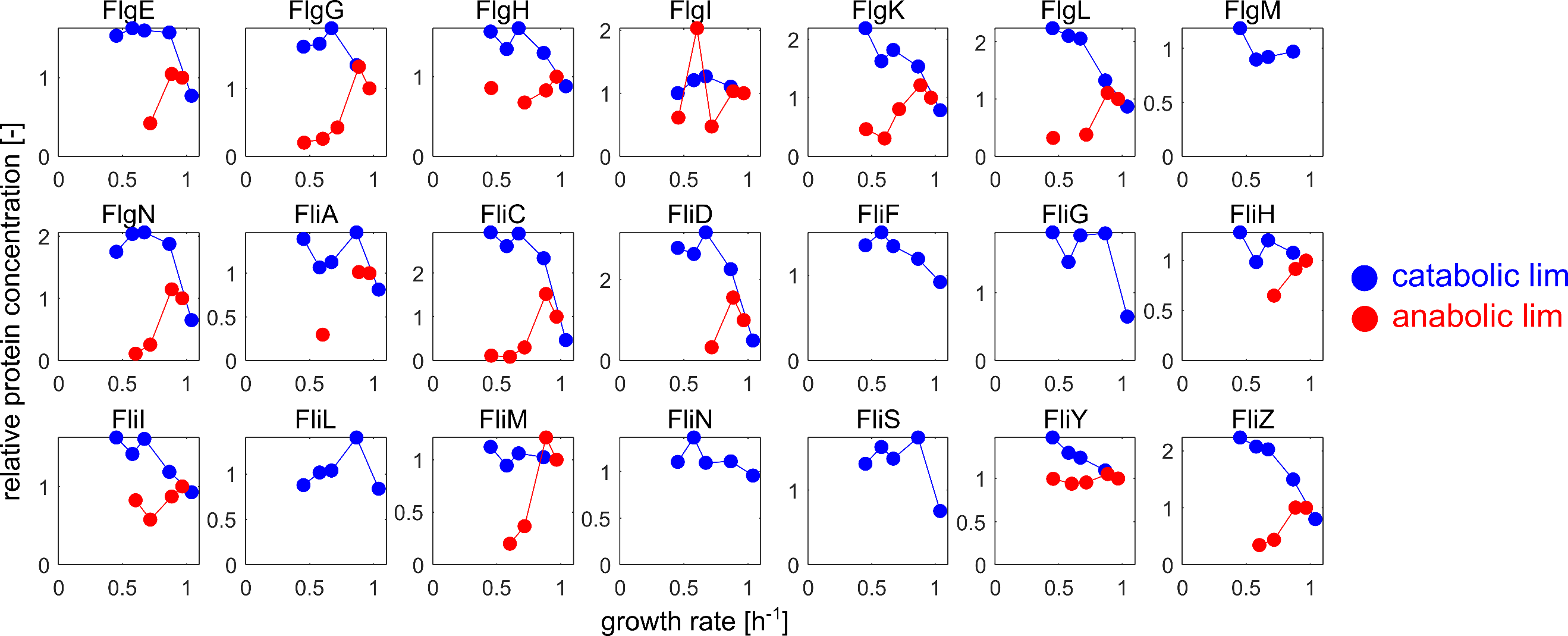


**Appendix Figure S19.** Relative concentration of selected flagella proteins in catabolic and anabolic limitations. Data from (Hui *et al*, 2015).

# References

Baba T, Ara T, Hasegawa M, Takai Y, Okumura Y, Baba M, Datsenko K a, Tomita M, Wanner BL & Mori H (2006) Construction of Escherichia coli K-12 in-frame, single-gene knockout mutants: the Keio collection. *Mol. Syst. Biol.* **2:** 2006.0008

Basan M, Hui S, Okano H, Zhang Z, Shen Y, Williamson JR & Hwa T (2015) Overflow metabolism in Escherichia coli results from efficient proteome allocation. *Nature* **528:** 99–104

Chubukov V, Uhr M, Le Chat L, Kleijn RJ, Jules M, Link H, Aymerich S, Stelling J & Sauer U (2013) Transcriptional regulation is insufficient to explain substrate-induced flux changes in Bacillus subtilis. *Mol. Syst. Biol.* **9:** 709

Daran-Lapujade P, Rossell S, van Gulik WM, Luttik MAH, de Groot MJL, Slijper M, Heck AJR, Daran J-M, de Winde JH, Westerhoff H V., Pronk JT & Bakker BM (2007) The fluxes through glycolytic enzymes in Saccharomyces cerevisiae are predominantly regulated at posttranscriptional levels. *Proc. Natl. Acad. Sci.* **104:** 15753–15758

Fuhrer T, Heer D, Begemann B & Zamboni N (2011) High-throughput, accurate mass metabolome profiling of cellular extracts by flow injection-time-of-flight mass spectrometry. *Anal. Chem.* **83:** 7074–80

Gerosa L, Haverkorn van Rijsewijk BRB, Christodoulou D, Kochanowski K, Schmidt TSB, Noor E & Sauer U (2015) Pseudo-transition Analysis Identifies the Key Regulators of Dynamic Metabolic Adaptations from Steady-State Data. *Cell Syst.* **1:** 270–282

Gerosa L, Kochanowski K, Heinemann M & Sauer U (2013) Dissecting specific and global transcriptional regulation of bacterial gene expression. *Mol. Syst. Biol.* **9:** 658

Hui S, Silverman JM, Chen SS, Erickson DW, Basan M, Wang J, Hwa T & Williamson JR (2015) Quantitative proteomic analysis reveals a simple strategy of global resource allocation in bacteria. *Mol. Syst. Biol.* **11:** e784–e784

Kochanowski K, Gerosa L, Brunner SF, Christodoulou D, Nikolaev Y V & Sauer U (2017) Few regulatory metabolites coordinate expression of central metabolic genes in Escherichia coli. *Mol. Syst. Biol.* **13:** 903

Kochanowski K, Volkmer B, Gerosa L, Haverkorn van Rijsewijk BR, Schmidt A & Heinemann M (2013) Functioning of a metabolic flux sensor in Escherichia coli. *Proc. Natl. Acad. Sci.* **110:** 1130–1135

Li G-W, Burkhardt D, Gross C & Weissman JS (2014) Quantifying Absolute Protein Synthesis Rates Reveals Principles Underlying Allocation of Cellular Resources. *Cell* **157:** 624–635

Link H, Buescher JM & Sauer U (2012) Targeted and quantitative metabolomics in bacteria. In *Systems Biology of Bacteria* pp 127–150. Elsevier Ltd

Link H, Kochanowski K & Sauer U (2013) Systematic identification of allosteric protein-metabolite interactions that control enzyme activity in vivo. *Nat. Biotechnol.* **31:** 357–361

Maaløe O (1979) Regulation of the Protein-Synthesizing Machinery—Ribosomes, tRNA, Factors, and So On. *Biol. Regul. Dev.* **1:** 487–542

Noor E, Flamholz A, Liebermeister W, Bar-Even A & Milo R (2013) A note on the kinetics of enzyme action: A decomposition that highlights thermodynamic effects. *FEBS Lett.* **587:** 2772–7

Rossell S, van der Weijden CC, Lindenbergh A, van Tuijl A, Francke C, Bakker BM & Westerhoff H V (2006) Unraveling the complexity of flux regulation: a new method demonstrated for nutrient starvation in Saccharomyces cerevisiae. *Proc. Natl. Acad. Sci. U. S. A.* **103:** 2166–71

Scott M, Gunderson CW, Mateescu EM, Zhang Z & Hwa T (2010) Interdependence of Cell Growth and Gene Expression: Origins and Consequences. *Science (80-. ).* **330:** 1099–1102

Soupene E, Van Heeswijk WC, Plumbridge J, Stewart V, Bertenthal D, Lee H, Prasad G, Paliy O, Charernnoppakul P & Kustu S (2003) Physiological studies of Escherichia coli strain MG1655: growth defects and apparent cross-regulation of gene expression. *J. Bacteriol.* **185:** 5611

You C, Okano H, Hui S, Zhang Z, Kim M, Gunderson CW, Wang Y-P, Lenz P, Yan D & Hwa T (2013) Coordination of bacterial proteome with metabolism by cyclic AMP signalling. *Nature* **500:** 301–306

Zaslaver A, Bren A, Ronen M, Itzkovitz S, Kikoin I, Shavit S, Liebermeister W, Surette MG & Alon U (2006) A comprehensive library of fluorescent transcriptional reporters for Escherichia coli. *Nat. Methods* **3:** 623–628
